# Deactivation of somatosensory and visual cortices during vestibular stimulation is associated with older age and poorer balance

**DOI:** 10.1371/journal.pone.0221954

**Published:** 2019-09-12

**Authors:** Fatemeh Noohi, Catherine Kinnaird, Yiri De Dios, Igor Kofman, Scott J. Wood, Jacob J. Bloomberg, Ajitkumar P. Mulavara, Kathleen H. Sienko, Thad A. Polk, Rachael D. Seidler

**Affiliations:** 1 Department of Kinesiology, University of Michigan, Ann Arbor, MI, United States of America; 2 Department of Psychology, University of Michigan, Ann Arbor, MI, United States of America; 3 Department of Biomedical Engineering, University of Michigan, Ann Arbor, MI, United States of America; 4 KBRwyle, Houston, TX, United States of America; 5 NASA Johnson Space Center, Houston, TX, United States of America; 6 Department of Applied Physiology & Kinesiology, University of Florida, Gainesville, FL, United States of America; Toronto Rehabilitation Institute - UHN, CANADA

## Abstract

Aging is associated with peripheral and central declines in vestibular processing and postural control. Here we used functional MRI to investigate age differences in neural vestibular representations in response to pneumatic tap stimulation. We also measured the amount of body sway in multiple balance tasks outside of the MRI scanner to assess the relationship between individuals’ balance ability and their vestibular neural response. We found a general pattern of activation in canonical vestibular cortex and deactivation in cross modal sensory regions in response to vestibular stimulation. We found that activation amplitude of the vestibular cortex was correlated with age, with younger individuals exhibiting higher activation. Deactivation of visual and somatosensory regions increased with age and was associated with poorer balance. The results demonstrate that brain activations and deactivations in response to vestibular stimuli are correlated with balance, and the pattern of these correlations varies with age. The findings also suggest that older adults exhibit less sensitivity to vestibular stimuli, and may compensate by differentially reweighting visual and somatosensory processes.

## Introduction

One third of older adults above age 65 years fall at least once per year [1], impacting quality of life and resulting in significant healthcare costs. Balance control is a complex sensorimotor-cognitive process, requiring perception and integration of visual, somatosensory, and vestibular inputs. Age effects on the vestibular system have been more extensively studied in recent years[2–4]. Several investigations have documented degeneration of vestibular neurons and hair cells with age. For example, Matheson et al. [5] reported that up to 40% of vestibular cells are lost by age 90. Moreover, neurons in the vestibular nuclei are lost at a rate of about 3% per decade after age 40 [6]. Despite these reports, studies have generally not reported associations between these peripheral vestibular changes and functional measures of balance.

Recent fMRI experiments have successfully mapped out central vestibular processing networks [7]. Two meta-analyses have shown that many brain regions are involved in vestibular processing, including the superior temporal gyri, inferior parietal cortex, middle frontal lobe, posterior cingulate, thalamus, and cerebellum [8,9]. The *parietal opercular* area OP2 and *retroinsular cortex* were the most commonly activated regions by different vestibular stimulation modes.

Central cortical responses to vestibular inputs are also affected by age [3]. Karim et al. [10] used an MR compatible force platform to simulate an active balance task while acquiring fMRI data. They found that older adults showed activation in the bilateral middle temporal gyri and fusiform gyri when performing the balance task inside the scanner. However, the authors only tested older adults in this study, precluding an analysis of age differences; in addition, the task likely engaged multiple sensory systems. In another study, the same group used fNIRS during upright balance assessments and reported that older adults exhibited greater bilateral activation in the superior temporal gyri in response to caloric vestibular stimulation in comparison to young adults [11]. Lin and colleagues [12] also used fNIRS to examine brain activity during upright balance tasks and found greater activation in occipital and frontal regions for older adults compared to middle aged adults. In contrast to these reports of age-related increases in cortical response, Cyran et al. [13] found that vestibular network functional connectivity (measured during galvanic vestibular stimulation) declines with age, and this decline is independent of changes in white matter microstructure measured with diffusion MRI. The authors also found that age differences in task-based vestibular connectivity were associated with a decrease in blood-oxygenation level dependent (BOLD) signal amplitude and an increase in its variability. However, they did not measure balance and thus the behavioral implications of their findings remain unclear.

Some studies have examined the functional impact of age differences in vestibular processing by correlating MRI measures of brain structure or function with balance measured outside of the scanner. For example, Sullivan et al. [14] showed that age-related increases in body sway were associated with increased white matter hyper-intensities and enlargement of the ventricles and sulci. Another study by Impe et al. [15] used diffusion MRI and showed that white matter integrity was predictive of postural stability when different sensory modalities were compromised during a sensory organization test, but only in older adults. Furthermore, Yuan et al. [16] showed that greater resting state functional connectivity (between sensorimotor, visual, vestibular, and left fronto-parietal cortical areas) is associated with higher walking speed in older adults (with the latter measured outside of the scanner). However, the authors did not provide a comparison between young and older adults.

Thus, the literature documents that older adults recruit more brain regions than young adults for balance and vestibular processing, while the connectivity of these regions is weaker than in young adults. This is generally similar to what has been reported for age differences in cognitive task performance [17,18] and motor task performance [19]. In general, previous reports of age differences in neural responses suggest a complex pattern of over- and under-activation [20,21]. Oftentimes the greater activation in older adults is interpreted as compensatory because it is correlated with better task performance [22,23]. However, greater neural activation in older adults has also been viewed as dedifferentiation when it is associated with poorer performance [18].

In the current study we used fMRI during central vestibular processing to examine age differences in neural activation and deactivation and their associations with balance abilities. We stimulated the vestibular system in the MRI scanner using a pneumatic tapper device (previously validated in Noohi et al. [24]) and measured postural stability outside of the scanner to test for associations between vestibular brain responses and balance with respect to age. Furman and Redfern [25] have suggested using alternative vestibular stimulation methods for older adults that are more sensitive and tolerable, since the current clinical methods are sometimes not sensitive enough to detect age differences. In our previous report with young adults [24] we found that low level pneumatic taps to the lateral cheekbones were well tolerated; moreover, activation in several brain regions was correlated with balance abilities. Here, we hypothesized that healthy older adults would show an overall increase in brain activation in response to vestibular stimulation compared to young adults. We also expected that the magnitude and pattern of vestibular neural responses would correlate with individual differences in postural stability and balance control.

## Materials and methods

### Participants

We recruited 15 healthy, right-handed older adults (65–80, $\bar{x}$ = 71.2 ± 4.14, 10 females) and 18 healthy, right-handed young adults (18–35, $\bar{x}$ = 21 ± 2.44, 8 females) from the University of Michigan M Health Research recruiting website. Subjects were screened prior to participation in the study and were excluded if they had any neurological, vestibular, or postural disorder or contraindications for MRI scanning. Individuals with scores of <26 on the Montreal Cognitive Assessment (MOCA) were excluded from the study [26]. The University of Michigan Medical Institutional Review Board approved the current study, and all subjects signed the written informed consent document prior to participation.

### Balance assessments

#### Center of Pressure (CoP) measures

Participants underwent a series of balance assessments on a force platform (AMTI Inc, USA) and center of pressure was captured via a Vicon motion capture system (Nexus, Vicon Inc). The balance tasks were categorized into four conditions: Romberg [feet placed together], tandem [feet placed heel to toe], normal stance, and single leg stance, with multiple levels of difficulty: eyes closed/open, still/yaw/pitch head movement, arms crossed/free, and on firm/compliant surface. Each of these conditions served to increase the difficulty of postural stability by removing or challenging a particular sensory input: closing the eyes removed the visual inputs, head movements challenged the vestibular system, crossing the arms served to prevent compensatory strategies, and the compliant surface challenged the somatosensory/tactile system.

The yaw and pitch head movements consisted of sinusoidal movements (roughly ±20°, 0.6 Hz). Subjects were instructed to match their head movement to the beat of a metronome (0.6 Hz) that was used consistently for all subjects, within their comfort level. High-density viscoelastic foam was used as the compliant surface (length = 45cm, width = 45cm, thickness = 18cm; Natus Inc.). Subjects were instructed to take off their shoes before standing on the force platform and the viscoelastic foam. The order of the tasks was counterbalanced across subjects. Two spotters stood by the subjects to provide support in case of a fall.

The amount of body sway in these tasks reflected individual differences in postural stability. We calculated the amount of body sway as the area of an ellipse fit to the 95^th^ percentile confidence interval of center of pressure motion in the anterior-posterior and medial-lateral directions [27]. Better postural stability was reflected as less body sway and smaller ellipse size. The center of pressure measures have been validated in previous studies and shown to be highly correlated with the measures from Sensory Organization Tests [28].

For each task, we instructed subjects to maintain their balance for 30 seconds. If they lost their balance, the trial was stopped prematurely and was not included in the analysis. The measures of body sway were later used to assess the correlation between behavioral and neural metrics of vestibular function. Four tasks were selected for these correlation analyses:

1. Normal (feet apart) stance on firm surface with eyes open (baseline measure, reliance on visual, vestibular and somatosensory inputs)
2. Romberg (feet together) stance on firm surface with eyes closed (removed visual inputs, reliance on vestibular and somatosensory inputs)
3. Romberg (feet together) stance on compliant surface with eyes closed (removed visual inputs, challenged somatosensory system, greater reliance on vestibular inputs)
4. Single leg stance (left foot) on firm surface with eyes open (challenged somatosensory system, greater reliance on visual and vestibular inputs)

These four tasks were selected because: 1) All subjects were able to complete these tasks for 30 seconds (this was not the case for other conditions); 2) Ruhe et al. [29] reviewed the reliability of CoP measures and found no specific task to be more reliable than the others; 3) these tasks represented a relatively step-wise increase in challenging the sensory modalities and balance maintenance; and 4) they were reported previously to be reliable predictors of fall in older adults [30–32].

#### Clinical tests of functional mobility

In addition to CoP measures, we administered two clinical tests of balance and gait: the modified Dynamic Gait Index (mDGI) [33] and the Timed Up and Go (TUG) test [34]. Both tests have high test-retest reliability [35].

Timed Up & Go (TUG): As described by Podsiadlo & Richardson [36], the TUG test requires subjects to stand up from a chair, walk for 3 meters, turn around, walk back to the chair, and sit down. The time for completing this task (under 20 seconds for healthy older adults) is considered an index of functional mobility and balance control. The TUG-manual task is a modified version of this task, in which subjects are performing the task while carrying a cup of water; we administered this version as well. Lower TUG scores (i.e. less time needed to finish the tests) indicate better functional mobility and balance.

Modified Dynamic Gait Index (mDGI): As described by Shumway-Cook et al. [37], the mDGI is a modified scoring index for the DGI, which evaluates three facets of walking performance: time, gait pattern, and level of assistance. This clinical measure indicates the ability of subjects to adjust their gait according to complex walking situations. The test consists of 8 tasks; the first is the baseline gait measure (low-challenged, self-paced). The other 7 tasks are designed to challenge the subject to respond to 4 different forms of environmental conditions (change in speed, change in direction, obstacle avoidance, & climbing stairs). Higher mDGI scores indicate better ability to maintain balance.

### Functional Magnetic Resonance Imaging (fMRI)

The fMRI acquisition was conducted at the University of Michigan Functional MRI Laboratory, using a 3.0 T MRI scanner (General Electric Medical Systems, DISCOVERY MR750). The scanning protocol consisted of a high-resolution T1 structural scan (SPGR), a resting state connectivity scan, and fMRI during vestibular stimulation.

The structural MRI acquisition comprised a T1-weighted ascending sequential echo-planar scan (TR = 12.2 s, TE = 5.1 ms, FA = 15°, matrix size = 256 x256, FOV = 260 x260 mm, slice thickness = 1 mm, number of slices = 124) covering the whole brain and the cerebellum. A gradient-echo spiral sequence with ascending sequential slice ordering (FOV = 220 mm, TR = 2 s, TE = 30 ms, slice thickness = 1 mm, number of slices = 43, voxel size = 3.4375x3.4375mm) was used to acquire the functional images.

We minimized head movement using a Velcro strap over the forehead, and by placing foam padding on the left and right sides of the head. The padding also provided extra hearing protection (in addition to ear plugs) from the scanner noise. Furthermore, we placed a pulse oximeter over the subjects’ index finger and wrapped a respirometer belt around the subjects’ abdomen to collect physiological responses, which were later corrected by using the RETROICOR algorithm [38].

#### Stimulation of the vestibular system inside the scanner

We used a pneumatic skull tapper (MR compatible Pneumatic Tactile Pulse System (PnTPS), Engineering Acoustics Inc.) to stimulate the vestibular system inside the scanner [24]. The skull tapper delivered low force (19.6 N), compressed air taps (50–55 psi) to the lateral cheekbones, stimulating the otolith organs through bone conduction mechanisms [39–45]. We applied five stimulation trials on each side; each trial consisted of 24 taps delivered at 1Hz. This was implemented in a block design with five alternating periods of rest (20 seconds) and stimulation (24 seconds). The right and left side stimulations were administered in separate consecutive runs, approximately two minutes apart.

Before beginning each trial, the MRI technician reminded subjects to keep their eyes closed (to eliminate the effects of visual inputs on vestibular processing). After the completion of each trial, the MRI technician asked subjects to report the side of the stimulation to make sure they felt the taps on the correct spot. This also helped with keeping the subjects alert and communicative.

### Data analyses

#### Balance performance analysis

Center of Pressure (CoP) analysis: We used the Vicon software (Nexus, Vicon Inc) to analyze the center of pressure (CoP) data that were collected at 100 Hz on the force platform. The force platform channels were plugged into a 64 channel data acquisition board and the data were recorded using the Vicon Nexus software, which then automatically calculated CoP data from the raw channel data. The CoP data were then exported and outcome measures were calculated using Matlab. In keeping with previous studies [46,47], we applied a low pass filter with a 2^nd^ order recursive Butterworth filter with a cutoff of 10 Hz. For each balance trial, we fitted a 95% confidence interval ellipse to the anterior-posterior and medial-lateral trajectories. The area of the ellipse was calculated for correlation with individual differences in vestibular brain activity.

Functional Mobility tests: We recorded the time to complete the TUG and TUG-manual tests, and used the average performance time over three repetitions of each task. For the mDGI, we used the ordinal scaling system to score the subjects’ performance (with the highest possible score of 64).

#### fMRI data analyses

Preprocessing: We used SPM12 software (Welcome Department of Cognitive Neurology, London, UK [48]) to analyze the fMRI data. To ensure the steadiness of the MR signal, we discarded the first 10 seconds of each run. The University of Michigan Functional MRI Laboratory applies the RETROICOR algorithm [38] to the raw data to account for physiological responses (e.g. respiration and cardiac signals). We used the ARTifact detection toolbox [49] to detect any volumes with >2mm translational or >2° rotational movement; movement parameters were later used as covariates of no interest in the first level design matrix. Next, we applied slice-timing correction, and then co-registered the functional images with the anatomical image. The images were then normalized to the Montreal Neurological Institute (MNI152) template [48]. We normalized the cerebellar volumes separately using the Spatially Unbiased Infra-tentorial Template (SUIT) [50–53]. Next, we applied a Gaussian kernel function (8,8,8 mm) to spatially smooth the normalized images.

Comparing brain activity within and between young & older adult groups: The first level analysis was designed to compare the brain activity between rest and stimulation blocks, using the smoothed images. The resulting contrasts from the first level analysis were subsequently used in second level (group) analyses. We used SnPM13 software (http://warwick.ac.uk/snpm) to apply non-parametric permutation tests for statistical inferences [54]. We used the non-parametric approach due to the non-normal distribution of data in our sample. We conducted a two-sample t-test to test the hypothesis that brain activity in response to vestibular stimulation is higher in older adults compared to young adults. Further, we applied a simple regression to test the hypothesis that the higher brain activity is correlated with better balance performance. A false discovery rate (FDR) correction (p < .05) was applied to account for multiple comparisons.

Localization: The results from the above analyses were mapped to the Montreal Neurological Institute (MNI) atlas [48] to localize the corresponding brain regions, using the Harvard-Oxford maximum probability atlas of the bspmview toolbox[55] in SPM12. The cerebellar results were separately localized using the SUIT atlas.

Signal to Noise Ratio analysis: We calculated the fMRI signal to noise ratio, following the methods of Bernard et al. [56] in order to assess potential age differences in variability of vestibular activation.

Laterality Index analysis: We used the Laterality Index (LI) toolbox of SPM 12 [57,58] to assess whether there is a hemispheric dominance in vestibular processing, and whether that is influenced by age. We applied the bootstrapping method to minimize the potential effects of outliers, and performed the analysis on whole brain gray matter, superior temporal gyri, and the cerebellum. The analyses were conducted for each subject, and the LI scores were then compared between the age groups using a two-sample t-test.

Correlation analyses: Based on the results from the activation analyses, we created a combined region of interest (ROI) of all regions that were active during vestibular stimulation at an uncorrected P<0.001 in either young or older adults (i.e. the activation ROIs for the young adults and the old adults were calculated separately and then combined to create one set of ROIs). These ROIs were later used in a correlation analysis (with FDR correction level of p<0. 05) in order to find the regions of the brain where activity was associated with age and balance performance.

Correlation between age and brain activity measures: We used participants’ age as a covariate in an SnPM simple regression analysis to determine how brain activity varies with respect to age.

Correlation between balance measures and brain activity measures: We used the area of the ellipse, TUG, and MDGI scores as covariates in SnPM simple regression analyses to determine how brain activity varies with respect to measures of postural control.

Conjunction analyses: We performed conjunction analyses to identify regions of the brain where activity was associated with both age and balance performance. We applied the max function in the imcalc toolbox of SPM12, using the results of statistical inference from SnPM13 as the input images. A positive direction for conjunction of the two correlation analyses indicated that greater brain activity was associated with greater age and poorer balance (greater CoP and TUG measures/ smaller MDGI scores). A negative direction for the conjunction of the two correlation analyses indicated that greater brain activity was associated with younger age and better balance.

Correlation analyses for laterality index and signal to noise ratio: We conducted a simple regression to determine whether an individual’s balance measures were associated with their laterality index. Similarly, we conducted a simple regression to determine whether individual differences in postural control are associated with differences in signal to noise ratio.

## Results

### Balance assessment results

#### Center of Pressure (CoP) measures

**Fig 1:**
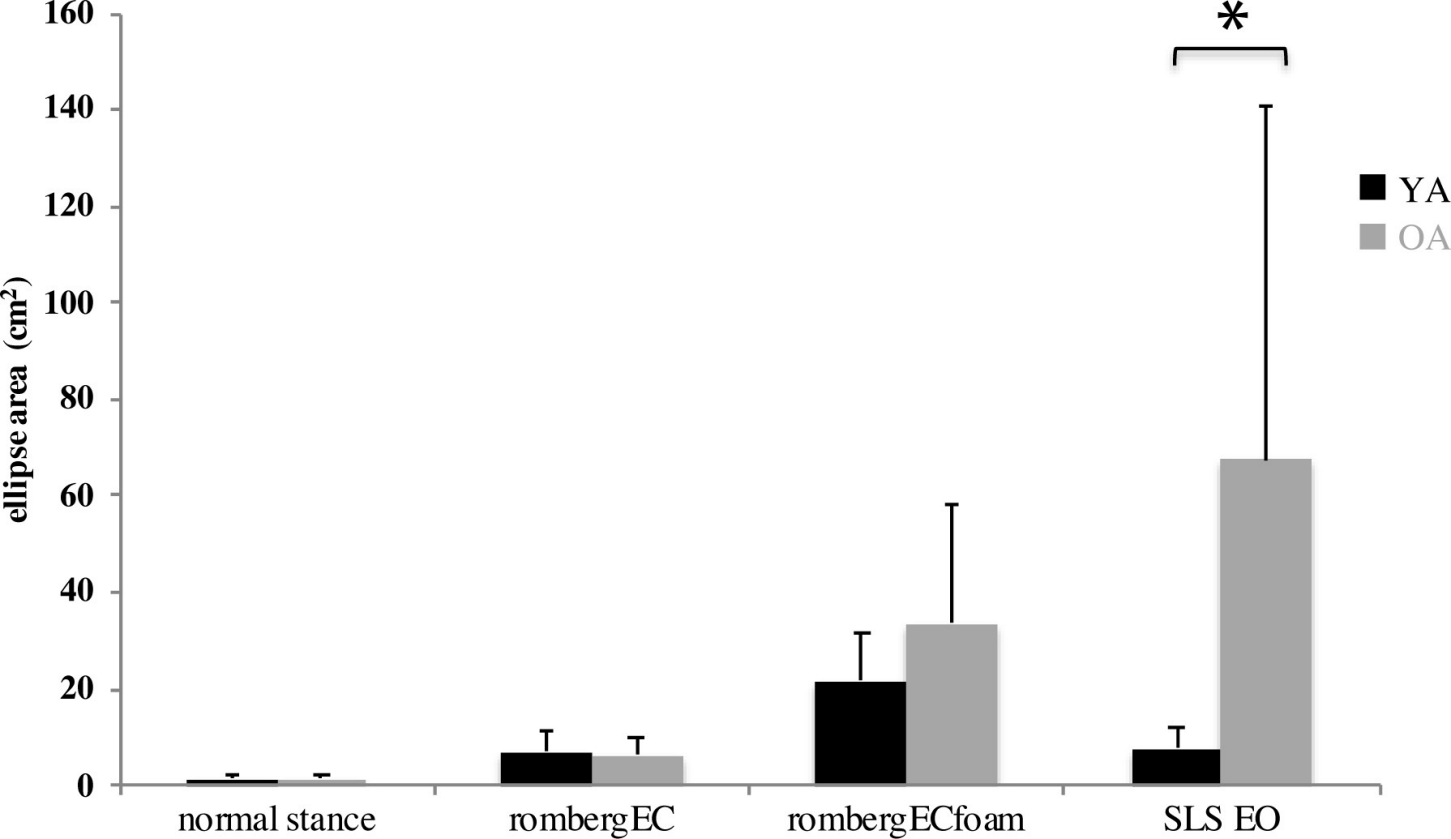
**Performance of young (black bars) and older (gray bars) adults in four balance tasks with different degrees of difficulty**. Task difficulty increases from left to right. Smaller ellipse area reflects less body sway (i.e. better balance). Error bars represent standard error. EO: Eyes Open, EC: Eyes Closed, SLS: Single Leg Stance. N = 12 Young, 14 Old.

We found a significant difference between young and older adults’ performance in single leg stance (SLS) with eyes open (t = -2.69, p = 0.01, df = 23, CI: -105.88, -13.95), documenting poorer balance performance (greater ellipse area) for older adults. The other CoP measures were not significantly different between young and older adults (Fig 1).

#### Clinical tests of functional mobility

**Fig 2:**
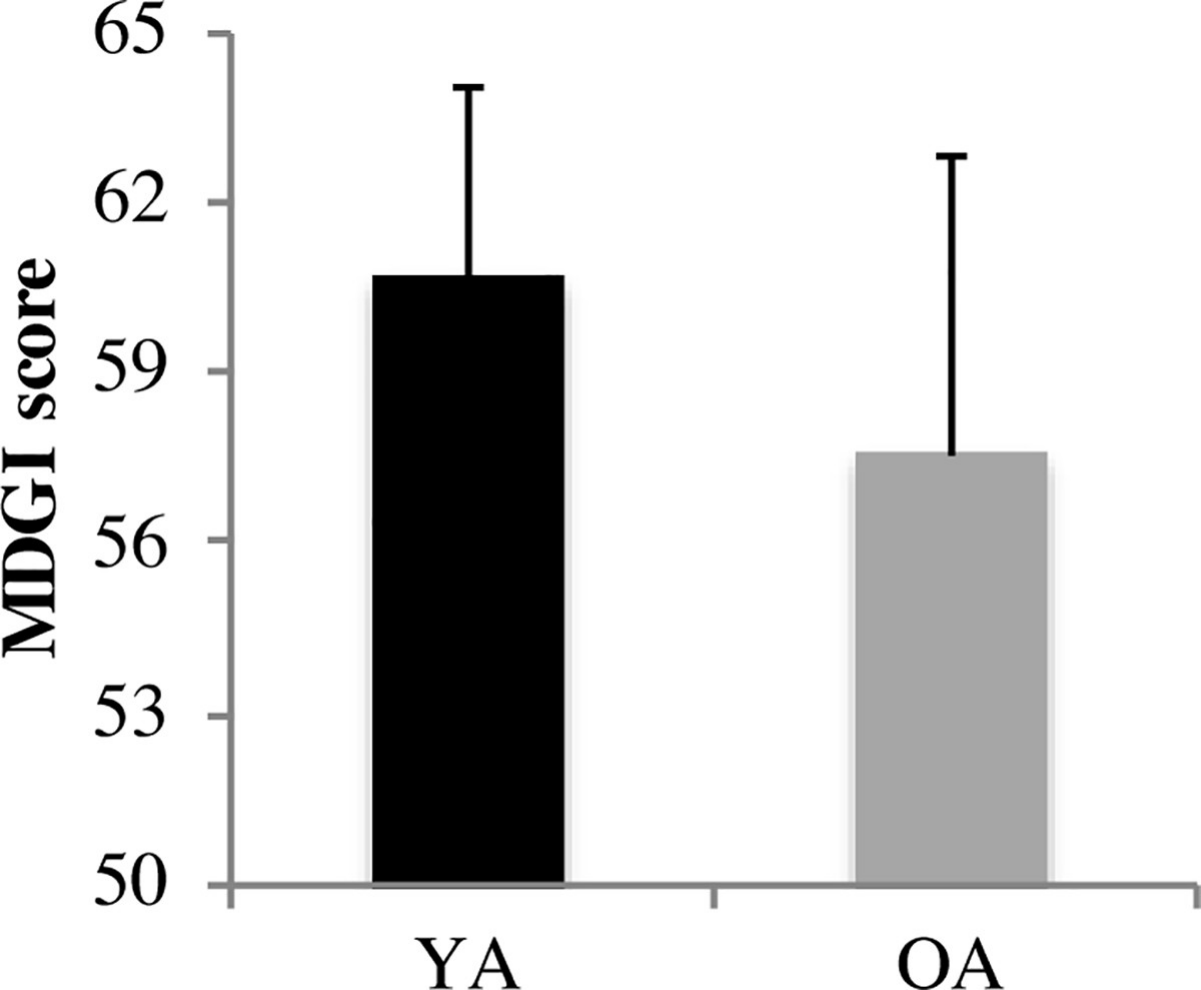
Performance of young and older adults in a series of gait assessment tasks (MDGI). Higher MDGI scores reflect better gait and balance. Error bars represent standard error. YA: Young Adults, OA: Older Adults; N = 6 YA, 14 OA.

**Fig 3:**
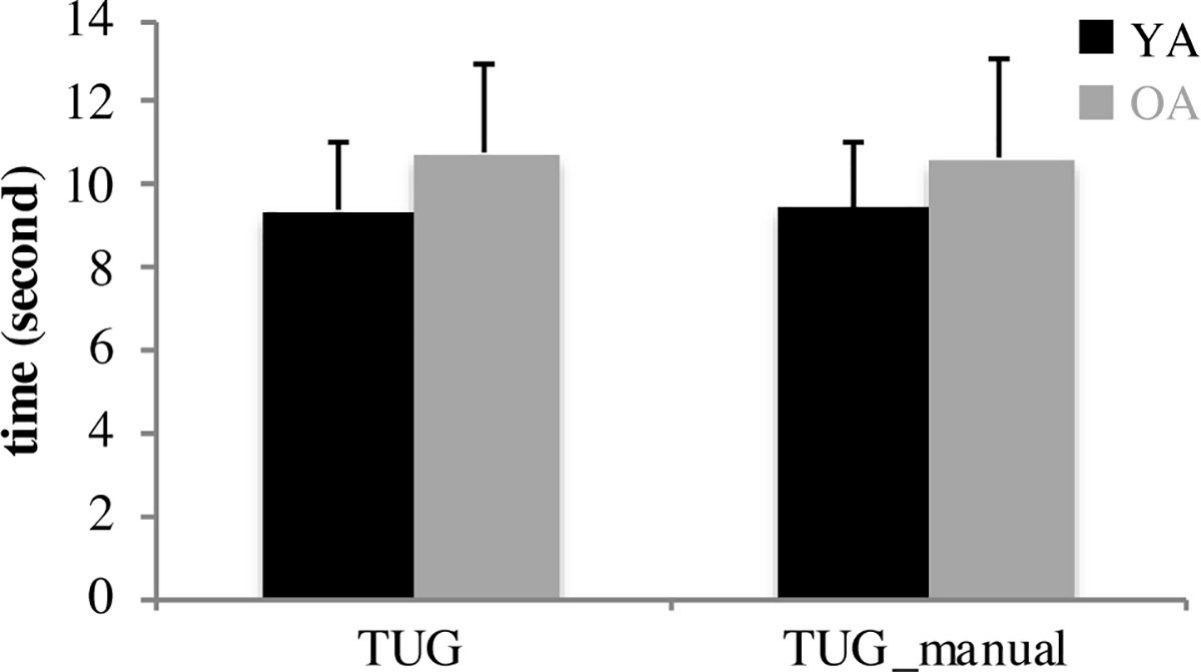
**Performance of young (black bars) and older (gray bars) adults in the TUG and TUG-manual tasks**. Lower scores reflect better gait and balance. Error bars represent standard error. YA: Young Adults, OA: Older Adults; N = 6 Young, 14 Old.

As shown in Figs 2 and 3, there was no significant difference between young and older adults’ MDGI and TUG scores.

### fMRI results

First we compared the activation patterns elicited by taps applied to the right and the left cheekbone, regardless of age. Not surprisingly given that a unilateral tap can result in stimulation of the vestibular system bilaterally, we found no significant difference between activity patterns or laterality index between the left and right tap conditions. Thus, to increase the power of our analyses, we pooled the two conditions together. Results are presented at p<0.05 with FDR correction, unless otherwise specified.

#### Comparing brain activity between young & older adult groups

There were no significant differences in brain activation or deactivation between the young versus old adults after the FDR p<0.05 corrections. The analyses conducted within the cerebellum after normalization to the SUIT template also showed no significant differences between young and older adults. We also found no significant difference in signal to noise ratio or in the laterality index of vestibular brain activity between young and older adults.

#### Associations between brain activity with age and balance

**Fig 4:**
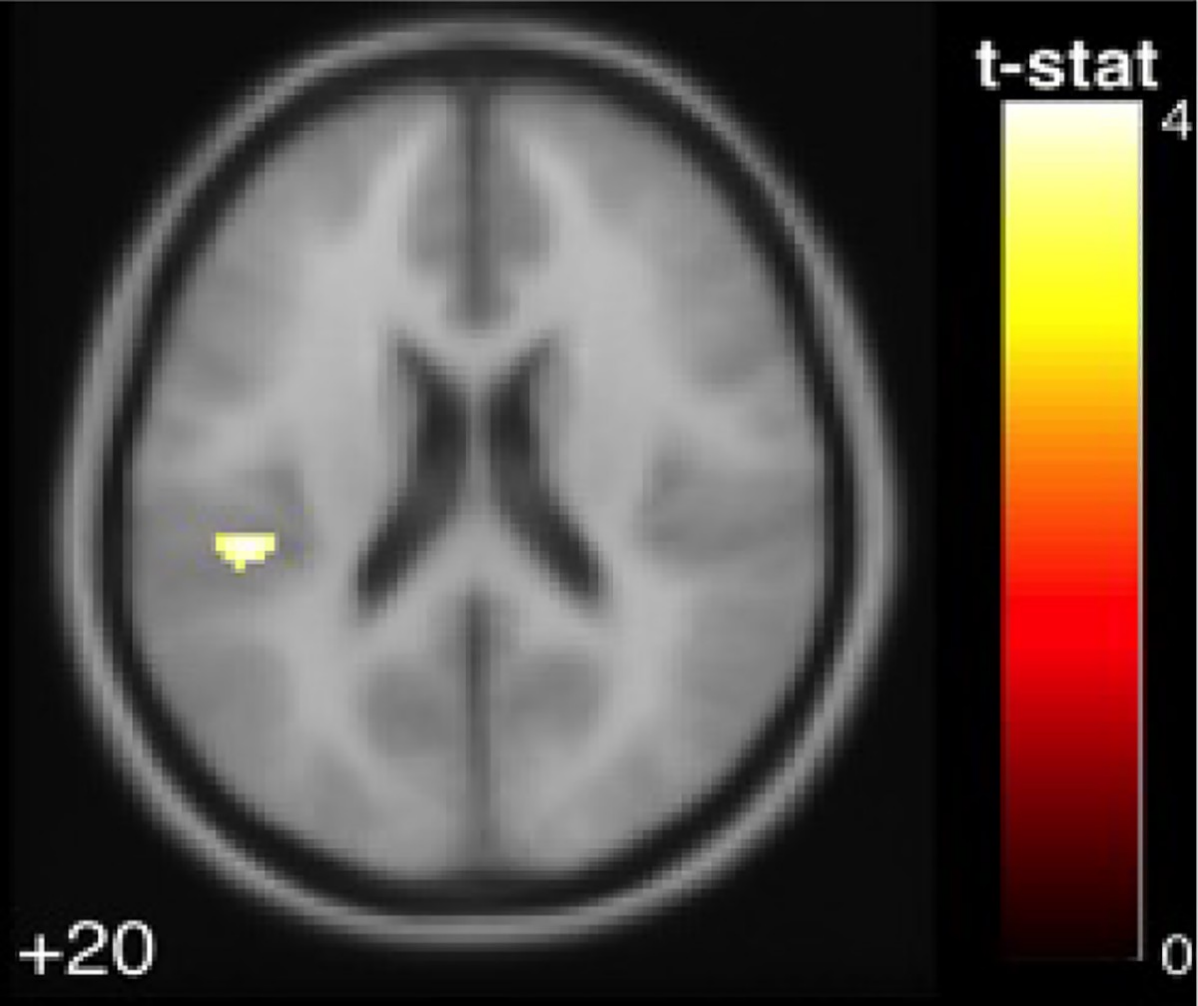
The parietal operculum cortex exhibited a negative association with age in the pooled young and older adults group. The colored voxels show brain regions in which the BOLD signal was significantly increased from rest to stimulation in a fashion that was correlated with subject age. The shade of the color in each brain map corresponds to the t-value in the legend bar.

**Fig 5:**
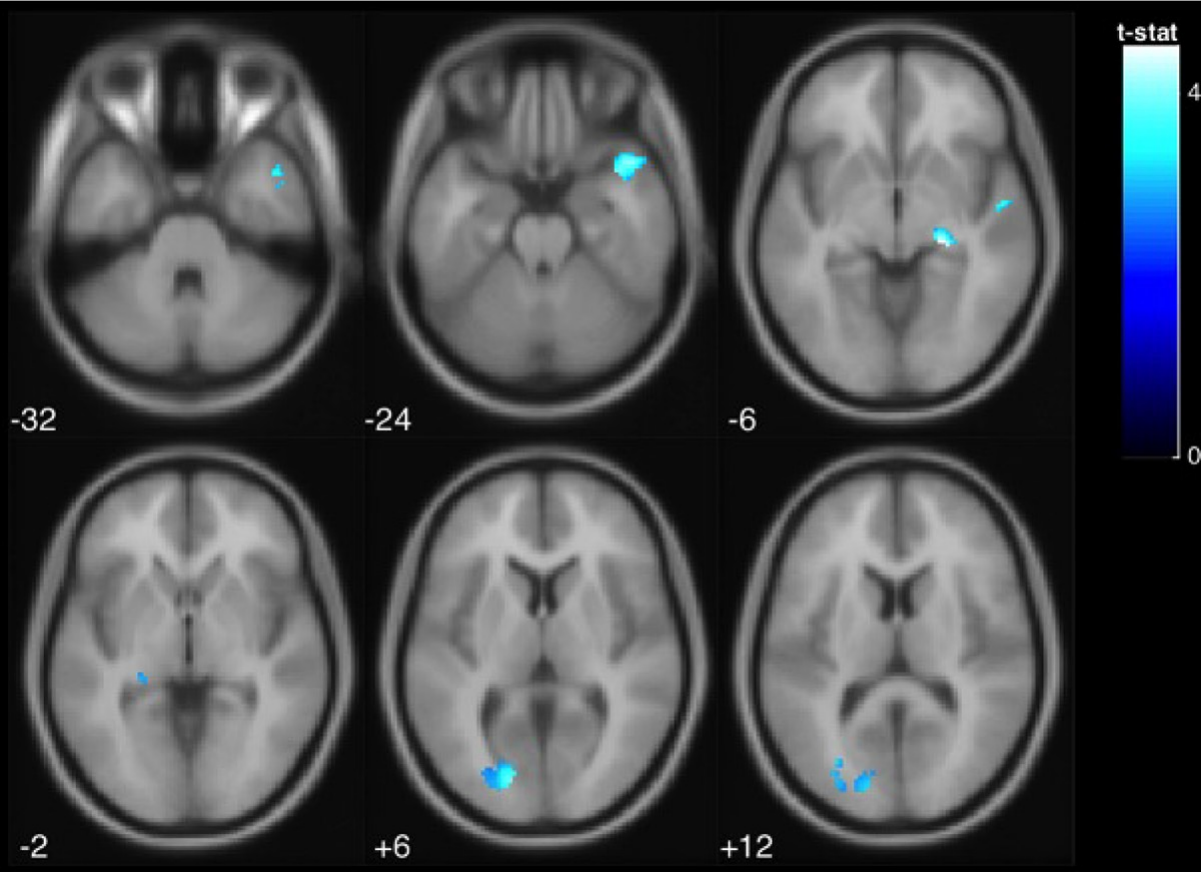
The intracalcarine cortex and temporal pole exhibited a positive association between age and deactivation level in the pooled young and older adults group. The colored voxels show the brain regions in which the BOLD signal was significantly decreased from rest to stimulation in a manner that was correlated with age. The shade of the color in each brain map corresponds to the t-value in the legend bar.

Correlation of brain function and age: There was a negative correlation between age and activation in the left parietal operculum (Fig 4, Table 1), with younger subjects exhibiting greater activation than older ones. Although this result did not survive FDR correction, it was significant at P<0.05 family-wise error correction (FWE). There was also a positive correlation between age and deactivation of the left intracalcarine cortex and the right temporal pole (the temporal pole has been shown to be functionally connected with somatosensory and visual networks [59], meaning that older adults deactivated these regions more than younger subjects (Fig 5, Table 2). Cerebellar activation and deactivation did not show any significant correlations with age.

**Table 1:** The activated region that exhibited a significant negative correlation with age (p < .05 FWE).

			MNI Coordinates		
Region Label	Extent	t-value	x	y	z
Parietal Operculum Cortex	40	4.096	-44	-30	20

**Table 2:** Deactivated regions that exhibited a significant positive correlation with age (p < .05 FDR).

			MNI Coordinates		
Region Label	Extent	t-value	x	y	z
Temporal Pole	221	4.083	46	14	-24
Intracalcarine Cortex	212	3.968	-16	-84	6
Anterior Superior Temporal Gyrus	16	3.623	54	-6	-6

**Fig 6:**
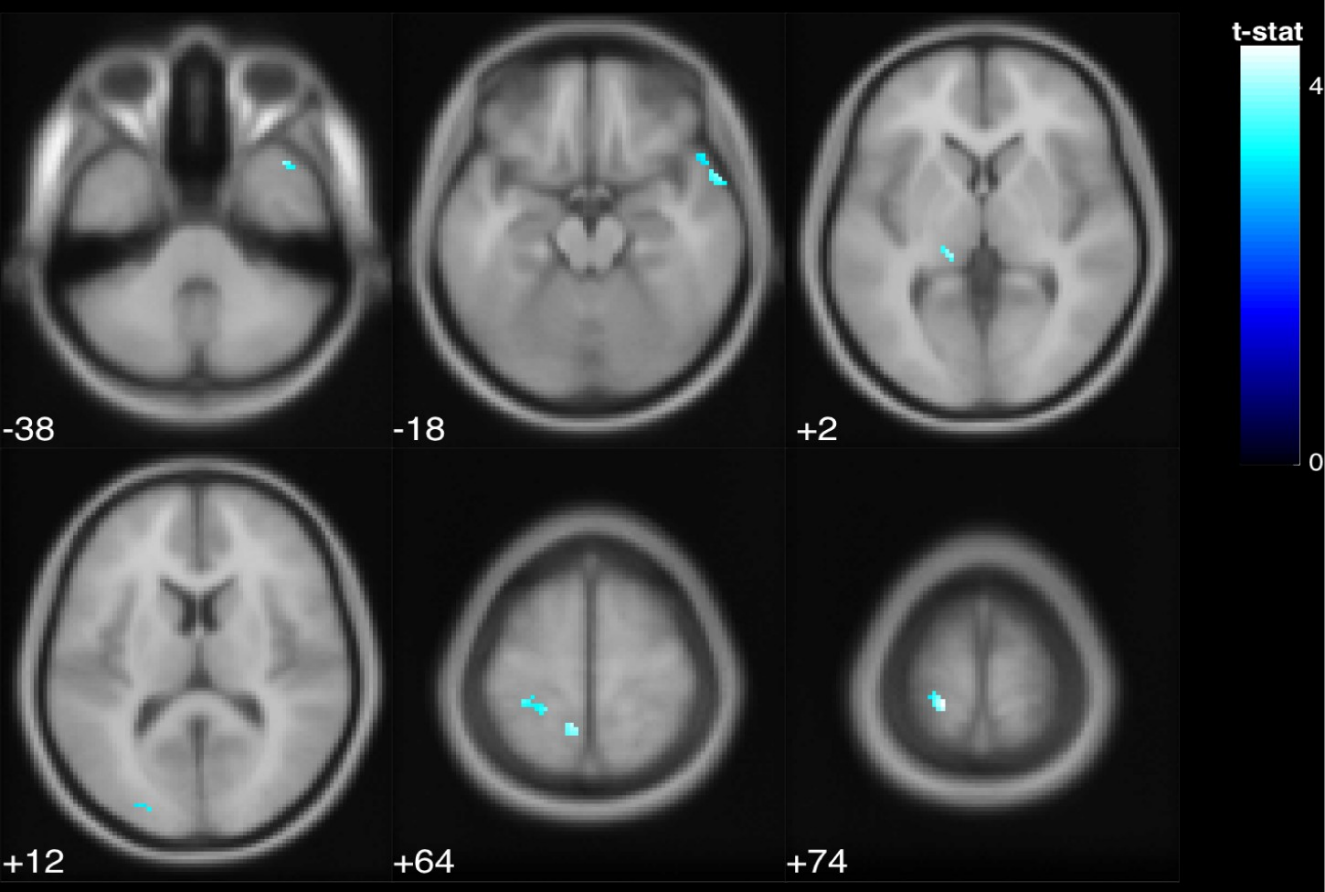
Regardless of age, greater deactivation of the postcentral gyrus and temporal pole was associated with poorer balance. The colored voxels show the regions of the brain in which the BOLD signal was significantly decreased from rest to stimulation in a way that was correlated with balance. The shade of the color in each brain map corresponds to the t-value in the legend bar.

Correlation of brain function and behavior: As shown in Fig 6, there was a positive correlation between balance performance in normal stance (indicated by area of ellipse) and deactivation of the postcentral gyri and temporal pole. In other words, participants with worse balance (a larger ellipse area during normal stance) deactivated these regions more, regardless of age (Table 3).

**Table 3:** Deactivated regions that exhibited a significant positive correlation with performance (p < .05 FDR).

Region Label	Extent	t-value	x	y	z
Postcentral Gyrus	121	4.452	-18	-38	74
Postcentral Gyrus	20	4.143	-8	-48	64
Temporal Pole	32	3.987	40	16	-38
Temporal Pole	48	3.938	60	8	-18
Lingual Gyrus	10	3.570	-30	-52	-4
Occipital Pole	12	3.328	-22	-90	12

Besides normal stance, no other balance measures were associated with cortical responses at FDR p< 0.05.

**Fig 7:**
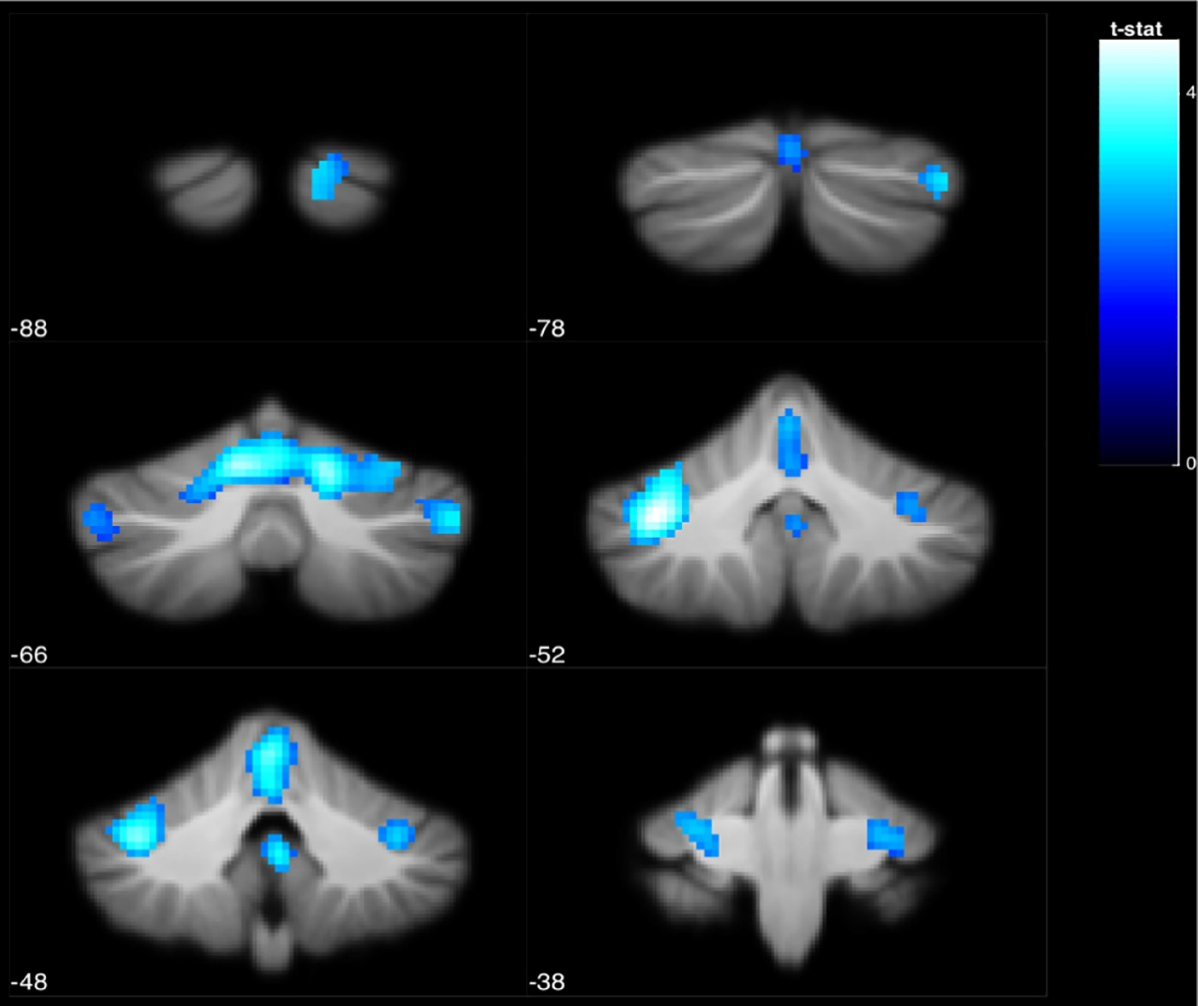
The brainstem and cerebellar lobule VI and Crus I exhibited a negative association between deactivation levels and balance performance in single leg stance (indicated by area of ellipse). The colored voxels show the regions of the brain in which the BOLD signal was significantly decreased from rest to stimulation in a way that was correlated with ellipse area across subjects. The shade of the color in each brain map corresponds to the t-value in the legend bar.

As for the cerebellar analyses, we found that performance in single leg stance was negatively correlated with deactivation of the brainstem, cerebellar lobule VI and Crus I. In other words, those who performed better in single leg stance exhibited greater deactivation of these regions in response to vestibular stimulation, regardless of age (Fig 7, Table 4).

**Table 4:** Deactivated regions that exhibited a significant negative correlation with performance (p < .05 FDR).

			MNI Coordinates		
Region Label	Extent	t-value	x	y	z
Crus I	2227	4.587	-36	-52	-35
Lobule VI	2227	4.401	16	-64	-23
Lobule VI	2227	4.289	-10	-64	-21
Brain-Stem	58	3.472	2	-48	-37
Crus I	200	3.381	50	-66	-37
Brain-Stem	90	3.274	-4	-22	-13
Crus II	98	3.143	14	-88	-31

Conjunction analyses: Next, we conducted a conjunction analysis to identify brain regions where activation or deactivation was commonly associated with both age and balance.

**Fig 8:**
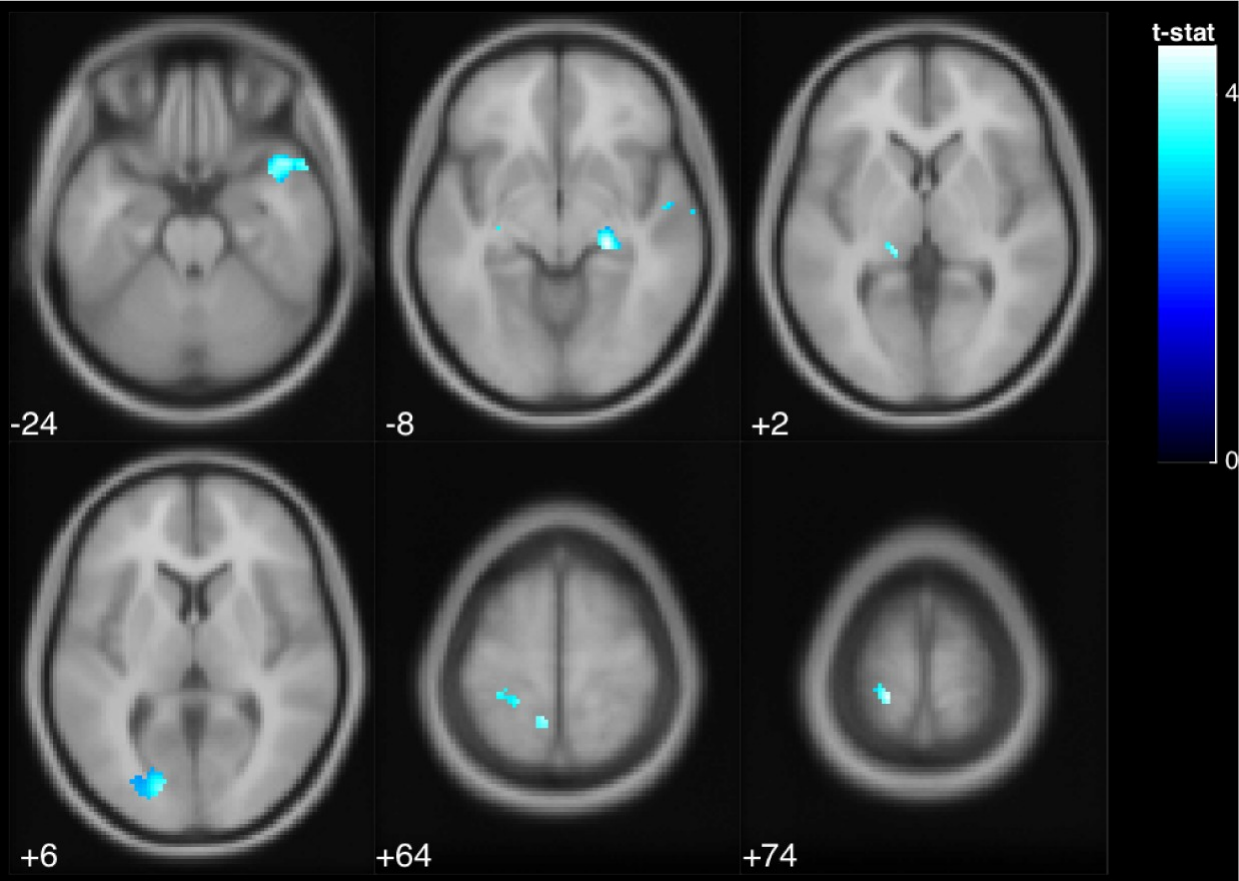
The deactivation of postcentral gyrus, intracalcarine cortex, and temporal pole was positively correlated with age and balance performance in normal stance (indicated by area of ellipse). The colored voxels show the regions of the brain in which the BOLD signal was significantly decreased from rest to stimulation in a way that correlated with age across subjects. The shade of the color in each brain map corresponds to the t-value in the legend bar.

We found that deactivation of left postcentral gyrus, intracalcarine cortex, and right temporal pole was positively correlated with both age and balance performance in normal stance (Fig 8); that is, those subjects who were older, and those subjects who performed more poorly in normal stance, deactivated these regions more than the other subjects (Table 5). None of the other whole brain or cerebellar conjunction analyses were significant. We found no significant associations between measures of balance and laterality index or signal to noise ratio.

**Table 5:** Deactivated regions that exhibited a significant positive correlation with age and performance (p < .05 FDR).

			MNI Coordinates		
Region Label	Extent	t-value	x	y	z
Postcentral Gyrus	121	4.452	-18	-38	74
Postcentral Gyrus	20	4.143	-8	-48	64
Temporal Pole	298	4.083	46	14	-24
Intracalcarine Cortex	215	3.968	-16	-84	6
Anterior Superior Temporal Gyrus	16	3.623	54	-6	-6
Lingual Gyrus	10	3.570	-30	-52	-4

## Discussion

In response to vestibular stimulation both young and older adults exhibited activation of the canonical vestibular cortex (i.e. bilateral parietal operculum). However, younger adults activated the vestibular cortex more strongly than older adults. In general, brain regions that process other sensory inputs (i.e. somatosensory cortex, visual cortex, and the cerebellum) exhibited deactivation, which—in contrast to the activation patterns—varied with age. The level of deactivation in these regions was correlated with both age and balance; as older age and poorer balance in the normal stance task were associated with greater deactivation of visual and somatosensory cortices and the temporal poles.

Consistent with these findings, other studies have documented similar cross modal sensory inhibition patterns [60]. For example, Bense et al. [61] showed that in response to galvanic vestibular stimulation, somatosensory and visual cortices were deactivated. Deutschlander et al. [62] demonstrated that with unimodal vestibular stimulation, visual cortex exhibited deactivation, and using unimodal visual stimuli resulted in deactivation of vestibular cortex. Laurienti et al. [63] reported that auditory stimulation resulted in activation of the auditory cortex and deactivation of the visual cortex; meanwhile the opposite pattern was observed in response to visual stimulation. Similarly, Uludağ et al. [64] used a flickering checkerboard to induce activation in the visual cortex, and found that simply closing the eyes resulted in deactivation of the visual cortex.

Relative reliance on vestibular, visual and somatosensory inputs varies within individuals. Studies have shown that, compared to young adults, older adults seem to rely more strongly on vision for balance control [65]. They also rely more on proprioception [66,67], which can be shifted towards vestibular reliance with active balance training [68,69]. Wiesmeier and colleagues [68] showed that balance training improved balance performance for older adults, and made them more reliant on vestibular inputs and less reliant on proprioceptive inputs, which was more similar to the pattern for young adults. Similarly, Bao et al. [69] showed that long-term balance training with sensory augmentation techniques resulted in improved postural control, with a significant increase in vestibular reliance.

We found that younger adults showed greater vestibular activity and older adults exhibited more deactivation of the somatosensory and visual cortices. It seems that when vestibular information is the most dominant sensory modality (as in our design), the suppression of other sensory systems (i.e. somatosensory and visual) enhances the allocation of neural resources in favor of vestibular processing, and more strongly in older adults. Greater deactivation in these regions was also an indicator of poorer balance in the normal stance task, in which all sensory modalities are available. Therefore, less engagement of visual and somatosensory cortices that occurs in response to unimodal vestibular stimulation is associated with poorer performance in this multisensory task. On the other hand, one might argue that these patterns of brain deactivation in correlation with balance should be interpreted in light of age differences in muscle response. As an adaptive strategy, older adults exhibit greater co-contraction of muscles in normal stance compared to young to maintain their balance [70]. One might speculate that older adults with better postural control may be better at implementing these adaptive strategies, and therefore exhibit less inhibition (i.e. higher recruitment of multiple regions, which is linked to muscle co-contraction). Moreover, our finding of overall stronger brain responses in young adults parallels previous reports [71,72]. For example, Burki et al. [73] showed an overall lower magnitude of brain activation in older adults performing an fMRI gait assessment task.

We also found that body sway was significantly higher in older adults when performing the single leg stance task. This task is designed to increase the difficulty of postural control by challenging the somatosensory system, so that vestibular and visual signals are weighted more strongly. As shown by Donath et al. [74], in a single leg stance with eyes open (and not the normal stance with eyes open) the ankle muscle coordination patterns are inverted in older adults and the muscle co-activation is doubled compared to young. Although we found a significant age difference in performance of this task, the association of performance and neural response did not vary with age. This could be potentially interpreted in light of the compensation-related utilization of neural circuits hypothesis (CRUNCH), proposed by Reuter-Lorenz & Cappell [17]. According to this theory, at lower task demands (i.e. normal stance task), older adults may compensate for their inefficient neural processing by engaging different brain regions compared to the young, which leads to behavioral performance equivalent to young. However, as the task difficulty increases (i.e. single leg stance), the available neural resources for older adults decline and the behavioral performance cannot be compensated for (hence the lack of age difference in neural recruitment patterns and significant age difference in performance).

Our current results also demonstrate that pneumatic skull taps elicit vestibular neural activation in older adults, while being tolerable, fast, and easy to conduct, as we reported previously in young adults [24]. Thus, this method could be implemented as a novel approach for studying vestibular function and its correlation with balance in a range of populations. By using fMRI we were able to assess the function of subcortical vestibular processing (e.g. in brainstem, cerebellum, and thalamus) in addition to cortical regions that would be captured with more portable neuroimaging techniques such as fNIRS.

### Limitations

The main limitation of the study is the relatively small sample size of both young and older adult groups; future studies with larger cohorts could verify the current findings. Another potential caveat of the study is that the stimulus was not adjusted for subjects’ individual threshold in sensory perception, as the main objective of the study was to assess the mean differences between two age groups. Also, as is typical with the skull tap method, the stimulus did not induce any vestibular percept, motion sensation, or movement in the position of the head. Instead of relying on subjective perception of the stimulus, the ocular Vestibular Evoked Myogenic Potential (oVEMP) assessment outside of the scanner was used to validate the successful stimulation of vestibular organs. Another caveat of the current study is the lack of a control condition to account for potential somatosensory/proprioceptive (i.e. tactile sensation of air puffs) response to the skull tapper. Nevertheless, no activation was found in somatosensory facial regions of the brain.

## Conclusion

In conclusion, we found that both age and balance performance are related to vestibular neural processing and deactivation of the somatosensory and visual cortices. Considering the high prevalence of falls among older adults, mortality of fall-related injuries, and costs of rehabilitation and healthcare [75], a better understanding of the aging vestibular system may lead to important, new approaches to balance interventions.
